# Multicenter Retrospective Study of Vascular Infections and Endocarditis Caused by *Campylobacter* spp., France

**DOI:** 10.3201/eid2903.221417

**Published:** 2023-03

**Authors:** Claire Tinévez, Philippe Lehours, Anne-Gaëlle Ranc, Yaniss Belaroussi, Fanny Velardo, Damien Dubois, Catherine Neuwirth, Hélène Pailhoriès, Marie Dorel, Genevieve Hery-Arnaud, Olivier Join-Lambert, Emmanuelle Gras, Stéphane Corvec, Cyrielle Codde, Damien Fournier, Hugo Boijout, Violaine Doat, Leslie Bouard, Anne-Sophie Lagneaux, Maxime Pichon, Célia Couzigou, Claire Letellier, Adrien Lemaignen, Emmanuelle Bille, Xavier Bérard, Caroline Caradu, Claire Webster, Didier Neau, Charles Cazanave, Mathilde Puges, the Campylobacteremia Study Group

**Affiliations:** Centre Hospitalier Universitaire de Bordeaux, Bordeaux, France (C. Tinévez, P. Lehours, Y. Belaroussi, X. Bérard, C. Caradu, D. Neau, C. Cazanave, M. Puges);; Centre Hospitalier Universitaire de Lyon, Lyon, France (A.-G. Ranc);; Institut National de la Santé et de la Recherche Médicale, Bordeaux (F. Velardo);; Centre Hospitalier Universitaire de Toulouse, Toulouse, France (D. Dubois);; Centre Hospitalier Universitaire de Dijon, Dijon, France (C. Neuwirth);; Centre Hospitalier Universitaire d’Angers, Angers, France (H. Pailhoriès);; Centre Hospitalier Universitaire de Rennes, Rennes, France (M. Dorel);; Centre Hospitalier Universitaire de Brest, Brest, France (G. Héry-Arnaud);; Centre Hospitalier Universitaire de Caen, Caen, France (O. Join-Lambert);; Hôpital Européen Georges–Pompidou, Paris, France (E. Gras);; Centre Hospitalier Universitaire de Nantes, Nantes, France (S. Corvec);; Centre Hospitalier Universitaire de Limoges, Limoges, France (C. Codde);; Centre Hospitalier Universitaire de Besançon, Besançon, France (D. Fournier);; Centre Hospitalier de Tarbes, Tarbes, France (H. Boijout);; Centre Hospitalier Pierre Oudot, Bourguoin-Jallieu, France (V. Doat);; Centre Hospitalier Départemental de Vendée, La Roche-Sur-Yon, France (L. Bouard);; Centre Hospitalier Universitaire de Nancy, Nancy, France (A.-S. Lagneaux);; Centre Hospitalier Universitaire de Poitiers, Poitiers, France (M. Pichon);; Centre Hospitalier de Rodez, Rodez, France (C. Couzigou);; Centre Hospitalier de Saint-Brieuc, Saint-Brieuc, France (C. Letellier);; Centre Hospitalier Universitaire de Tours, Tours, France (A. Lemaignen);; Centre Hospitalier de Necker-Enfants Malades, Paris (E. Bille);; Imperial College London, London, UK (C. Webster)

**Keywords:** *Campylobacter* spp., bacteria, bacterial infections, non-HACEK endocarditis, infectious aortitis, graft infections, thrombophlebitis, France

## Abstract

The incidence of campylobacteriosis has substantially increased over the past decade, notably in France. Secondary localizations complicating invasive infections are poorly described. We aimed to describe vascular infection or endocarditis caused by *Campylobacter* spp. We included 57 patients from a nationwide 5-year retrospective study on *Campylobacter* spp. bacteremia conducted in France; 44 patients had vascular infections, 12 had endocarditis, and 1 had both conditions. *Campylobacter fetus* was the most frequently involved species (83%). Antibiotic treatment involved a β-lactam monotherapy (54%) or was combined with a fluoroquinolone or an aminoglycoside (44%). The mortality rate was 25%. Relapse occurred in 8% of cases and was associated with delayed initiation of an efficient antimicrobial therapy after the first symptoms, diabetes, and coexistence of an osteoarticular location. Cardiovascular *Campylobacter* spp. infections are associated with a high mortality rate. Systematically searching for those localizations in cases of *C. fetus* bacteremia may be warranted.

Campylobacteriosis is the leading cause of foodborne bacterial gastroenteritis. Its incidence in North America, Europe, and Australia is alarming, and data from Africa, Asia, and the Middle East indicate that campylobacteriosis is endemic in several areas (*1*–*3*). The incidence of campylobacteriosis seems to have increased over recent years but might partially be overestimated because of differences in molecular techniques.

*Campylobacter* spp. is a gram-negative mobile curved rod. After digestive contamination, it can translocate through the gastrointestinal barrier, leading to bacteremia. This complication is poorly described because of its scarcity, accounting for <1% of *Campylobacter* spp. infections but having substantial mortality rates (3%–28%) (*4*–*6*). Bacteremias can be complicated by secondary localizations in the joints, bones, soft tissues, arterial wall, and valves (*5*,*7*,*8*). Lack of awareness of this risk and a challenging diagnosis caused by tedious culture may be responsible for underdiagnosis.

Vascular infections and endocarditis caused by *Campylobacter* spp. have been poorly described in the literature; therefore, clinical manifestations, treatment, and outcomes remain unclear. Identifying the predisposing underlying conditions for *Campylobacter* spp. vascular infections or endocarditis and recognizing evocative clinical and biologic signs could lead to an earlier effective antibiotic therapy. Our study aimed to describe *Campylobacter* spp.–related vascular infections and endocarditis in France and analyze the factors associated with 3-month mortality rates.

## Methods

### Study Design and Patients

We conducted an ancillary study from the Campylobacteremia Project (*6*), a multicenter retrospective study conducted in 37 hospitals participating in the surveillance network of France’s National Reference Centre for Campylobacters and Helicobacters (NRCCH), along with other hospitals in France. The Campylobacteremia study included all patients with *Campylobacter* spp. bacteremia during January 1, 2015–December 31, 2019. We extracted and analyzed records from patients with vascular localizations or endocarditis for our study. We also included patients with *Campylobacter* spp. identification from a retrospective cohort of vascular infections in Bordeaux University Hospital (BUH; Bordeaux, France) during January 1, 2004–December 31, 2019, excluding patients already included through the NRCCH.

### Data Collection

We retrospectively extracted data on demographic characteristics, clinical signs, underlying conditions previously described as risk factors of campylobacteriosis, or cardiovascular infections (*4*–*10*) and medico-surgical treatment from medical records through a standardized questionnaire sent to clinicians and microbiologists. We also extracted microbiologic data, especially identification to species level, results of concomitant stool or any other site culture (e.g., fluid and biopsy), and susceptibility to ampicillin, amoxicillin/clavulanic acid, erythromycin, tetracyclines, gentamicin, fluoroquinolones, and imipenem when tested.

### Definitions

We defined endovascular localizations by a positive vascular biopsy, graft, blood culture (or a combination of these) and evocative images on computed tomography, ^18^F-fluoro-deoxyglucose-positron emission tomography/computed tomography (^18^F-FDG PET/CT), or leukocyte scan based on the American Heart Association consensus for native infections and Management of Aortic Graft Infection Collaboration (MAGIC) criteria for vascular graft and endograft infections (VGEIs) (*10*,*11*). We defined endocarditis by a positive valvular biopsy, blood culture, or both, associated with evocative images on echocardiography, ^18^F-FDG PET/CT, or leukocyte scan according to the European Society of Cardiology 2015 modified criteria for diagnosing infective endocarditis (*9*).

We considered antibiotic treatment appropriate if the strain was susceptible to >1 of the drugs prescribed, according to the Antibiogram Committee of the French Society of Microbiology and European Committee On Antimicrobial Susceptibility Testing recommendations (*12*). *Campylobacter* spp. are naturally resistant to third-generation cephalosporins, ticarcillin, and piperacillin, so we considered those antibiotics to be inappropriate. 

We defined relapse by >1 new positive blood culture with *Campylobacter* spp. after clinical sign resolution and apyrexia or negative control blood culture. We defined 3-month mortality as death within 3 months of the first positive blood culture.

### Microbiological Diagnosis

All participating laboratories used continually monitored noninvasive blood culture systems (e.g., BacT/Alert and Virtuo [bioMérieux, https://www.biomerieux.com] or Bactec [Becton Dickinson, https://www.bd.com]). Each blood culture set included an aerobic and an anaerobic bottle inoculated with 10 mL of blood and incubated for 5 days. Two sets of blood culture were recommended. We performed Gram staining and fresh examinations for positive samples. We identified curved or spiral-shaped gram-negative rods as *Campylobacter* spp. We inoculated a blood agar plate and incubated it in a microaerobic atmosphere (6% O_2_, 7% CO_2_, 7% H_2_, and 78% N_2_) at 35°C. For patients who underwent vascular surgery, we obtained several samples from vascular tissue, thrombus, or grafts; for patients who underwent valvular surgery, we analyzed native or prosthetic valves. We plated intra-operative samples onto polyvitex chocolate agars (bioMérieux) and inoculated them into 10 mL of Schaedler and brain–heart broth. We incubated agar plates at 37°C for 14 days in aerobic atmosphere with 5% CO_2_ and for 14 days in anaerobic atmosphere. We incubated broth at 37°C for 15 days and subsequently plated cloudy broth media on polyvitex chocolate agar plates and incubated them in a 5% CO_2_ atmosphere for 7 days. We performed bacterial identification by using matrix-assisted laser desorption/ionization time-of-flight mass spectrometry (*13*) from positive standard bacterial culture. We interpreted susceptibility testing according to Antibiogram Committee of the French Society of Microbiology and European Committee On Antimicrobial Susceptibility Testing recommendations (*12*).

### Objectives

Our primary objective was to evaluate the risk factors of 3-month mortality in patients with *Campylobacter* spp. vascular infection, endocarditis, or both. The secondary objectives were to describe the epidemiology, clinical manifestations, and therapeutic management and to evaluate risk factors of relapse.

### Ethics Approval

We declared our study to France’s National Institute of Health Data (https://www.snds.gouv.fr). We reported our retrospective cohort with France’s data protection authority (https://www.cnil.fr).

### Statistical Analysis

We expressed descriptive statistics as percentages for categorical variables and as the mean with SD and median with interquartile range (IQR) for continuous variables. We performed univariate analyses using Fisher exact test for count data, Wilcoxon test, and Pearson χ^2^ test with Yates’ continuity correction to identify the factors associated with a fatal outcome within 3 months. We considered results with p values <0.05 to be statistically significant. We performed statistical analyses with R studio version 1.2.5033 (https://rstudio.com).

## Results

**Figure 1:**
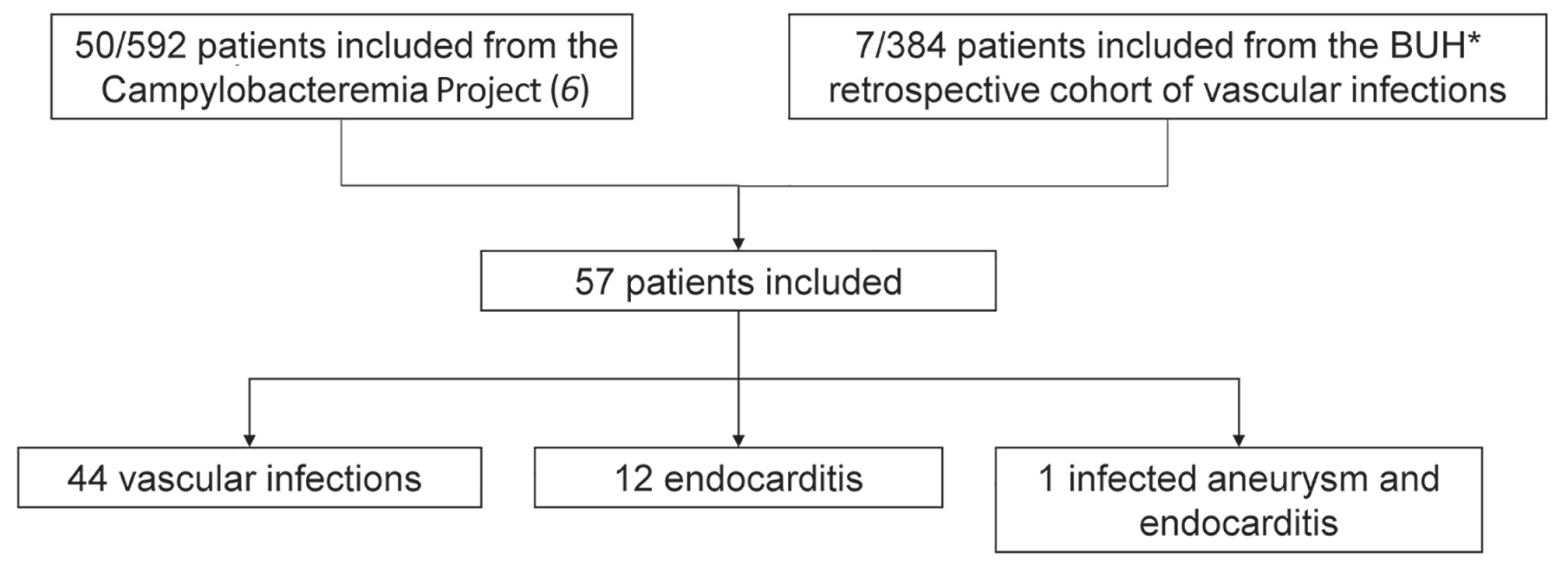
Flowchart of 57 patients with *Campylobacter* spp. vascular infections in a multicenter retrospective study on vascular infections and endocarditis caused by *Campylobacter* spp., France. BUH, Bordeaux University Hospital (Bordeaux, France).

Among 592 patients with *Campylobacter* spp. bacteremia, 57 were included in this analysis (Figure 1); 38 had a vascular infection (6.6%) and 12 had endocarditis (2.1%). Seven more patients among the 384 included in the BUH retrospective cohort before 2015 or without bacteremia were included. Overall, most included patients had a vascular infection (n = 44), followed by endocarditis (n = 12). One had both endocarditis and an infectious native aortic aneurysm.

### Demographic Data and Clinical Characteristics of Vascular Infection Cases

We compared clinical characteristics of patients with native (n = 30) or prosthetic (n = 15) vascular infections (Table 1). Male (80%) and elderly (64.9% were >65 years of age) patients were predominantly affected. Most patients had underlying conditions, mainly cardiovascular, and impairing immunity conditions; 26.7% were active smokers, 24.4% had diabetes, 24.4% had a history of aortic aneurysm, and 22.2% had ischemic cardiomyopathy.

**Table 1:** Characteristics of 45 patients with *Campylobacter* spp. vascular infections in a multicenter retrospective study on vascular infections and endocarditis caused by *Campylobacter* spp., France*

Characteristic	Vascular infection	Native vascular infection	Vascular graft or endograft infection
All patients	45 (100)	30 (66.7)	15 (33.3)
Age, y, median (interquartile range)	69.5 (61.2–81.3)	69.5 (61.8–81.3)	70 (62–81)
Sex			
M	36 (80)	23 (76.7)	13 (86.7)
F	9 (20)	7 (23.3)	2 (13.3)
Localization			
Aortic	30 (66.7)	17 (56.7)	13 (86.7)
Peripheral artery	7 (15.6)	6 (20)	1 (6.7)
Venous involvement	5 (11.1)	4 (13.3)	1 (6.7)
Lymphatic involvement	1 (2.2)	1 (3.3)	0
Not available	2 (4.4)	2 (6.7)	0
Underlying condition			
Preexisting aortic aneurysm	11 (24.4)	9 (30)	2 (13.3)
Ischemic cardiomyopathy	10 (22.2)	8 (26.7)	2 (13.3)
Tobacco use	12 (26.7)	8 (26.7)	4 (26.7)
Chronic liver disease	5 (11.1)	4 (13.3)	1 (3.3)
Diabetes	11 (24.4)	8 (26.7)	3 (20)
Chronic renal failure	9 (20)	5 (16.7)	4 (26.7)
Hematologic malignancy	2 (4.4)	2 (6.7)	0
Solid neoplasm	11 (24.4)	6 (20)	5 (33.3)
Immunodeficiency	7 (15.6)	6 (20)	1 (6.7)
Clinical manifestations†			
Fever	32 (71.1)	24 (80)	8 (53.3)
Septic shock	1 (2.2)	1 (3.3)	0
Hemorrhagic shock	4 (8.9)	3 (10)	1 (6.7)
Diarrhea	10 (22.2)	6 (20)	4 (26.7)
Gastrointestinal bleeding	6 (13.3)	5 (16.7)	1 (3.3)
Abdominal or lumbar pain	23 (51.1)	16 (53.3)	7 (46.7)
Acute limb ischemia	3 (6.7)	3 (10)	0
Osteoarticular involvement	4 (8.9)	2 (6.7)	2 (13.3)
*Campylobacter* species			
* C. fetus*	36 (80)	23 (76.7)	13 (86.7)
* C. jejuni*	5 (11.1)	4 (13.3)	1 (3.3)
Other *Campylobacter* spp.	4 (8.9)	3 (10)	1 (3.3)

*Values are no. (%) except as indicated. †Signs and symptoms occurring within the past month were considered.

Fever (71.1% of cases) and abdominal or lumbar pain (51.1% of cases) were the most common clinical signs. Diarrhea was quite rare (22.2%).

The infections were heterogeneous. The aorta was the most commonly infected vessel (66.7%); however, peripheral arteries could also be involved, either iliac (n = 2), popliteal (n = 3), gastroduodenal (n = 1), or carotid (n = 1). Some rare venous infections were described, either portal veins (n = 2), jugular-peritoneal shunt (n = 1), sural or femoral veins (n = 1 each), and finally a lymphangioma of the lower limb (n = 1). One third of these cases occurred on vascular grafts or endografts (33.3%), including 13 aortic, 1 femoro-popliteal, and 1 jugular-peritoneal shunt.

Four of these patients also had an osteoarticular infection, 1 had hip arthritis, and 3 had spondylodiscitis, 2 of which occurred in patients with aortitis, suggesting a contiguous infection. One of these 2 patients also had a psoas abscess.

### Demographic Data and Clinical Characteristics of Endocarditis Cases

We compared clinical characteristics of patients with endocarditis (Table 2). Again, the clinical manifestations were nonspecific; most patients were febrile (84.6%), and a cardiac murmur was found in only 4 patients.

**Table 2:** Characteristics of 13 patients with *Campylobacter* spp. endocarditis in a multicenter retrospective study on vascular infections and endocarditis caused by *Campylobacter* spp., France*

Characteristic	Infective endocarditis	Native valve infective endocarditis	Prosthetic valve infective endocarditis and CIED infection
All patients	13 (100)	4 (30.8)	9 (69.2)
Age, y, median (interquartile range)	67 (60.3–80.8)	64 (57–80)	67 (60.3–80.8)
Sex			
M	13 (100)	4 (30.8)	9 (69.2)
F	0	0	0
Localization			
Aortic valve	9 (69.2)	3	6
Mitral valve	2 (15.4)	1	1
CIED	2 (15.4)	0	2
Underlying condition			
Ischemic cardiomyopathy	2 (15.4)	0	2
Chronic liver disease	2 (15.4)	1	1
Diabetes	4 (30.8)	2	2
Chronic renal failure	4 (30.8)	2	2
Hematologic malignancy	1 (7.7)	0	1
Solid neoplasm	2 (15.4)	1	1
Immunodeficiency	4 (30.8)	1	3
Clinical manifestations†			
Fever	11 (84.6)	3	8
Septic shock	2 (15.4)	1	1
Diarrhea	3 (23.1)	1	2
Gastrointestinal bleeding	1 (7.7)	0	1
Abdominal or lumbar pain	3 (23.1)	2	1
Cardiac murmur	4 (30.8)	1	3
Cardiac failure	1 (7.7)	1	0
*Campylobacter* species			
* C. fetus*	12 (92.3)	4	8
* C. jejuni*	1 (7.7)	0	1

*Values are no. (%) except as indicated. CIED, cardiac implantable electronic device. †Signs and symptoms occurring within the past month were considered.

Valvular infections mostly occurred on the aortic valve (n = 9), and only 2 were on the mitral valve. No right-sided infective endocarditis was found. Those infections involved 7 prosthetic valves, 4 native valves, and 2 intracardiac devices (pacemakers). The time interval between valve or pacemaker implantation and endocarditis was >1 year in all cases.

### Diagnostic Imaging Results

All but 1 of the patients with vascular infection had documented imaging procedure data (Table 3). Detailed echography data were reported in 11 cases of endocarditis. All of them had major criteria for endocarditis, either typical oscillating valvular vegetation (n = 11), cardiac abscess (n = 3), valve perforation (n = 1), or prosthetic valve dehiscence (n = 1). One diagnosis was made on ^18^F-FDG PET/CT, which revealed hypermetabolism around the site of a prosthetic aortic valve associated with a thoracic aorta aneurysm. The last case, a mediastinitis associated with pacemaker infection, was diagnosed intraoperatively.

**Table 3:** Imaging results for 44 patients with vascular infections in a multicenter retrospective study on vascular infections and endocarditis caused by *Campylobacter* spp., France*

Radiologic finding	Native vascular infection, no.	Vascular graft and endograft infection, no.
All patients	30	14
Computed tomography	23	12
Perivascular or graft infiltration	5	5
Perivascular or graft gas	2	2
Abscess	1	4
Dissection	2	0
Pseudoaneurysm	3	2
Rupture	6	2
Thrombosis	4	3
Enteric contact with aorta	1	2
^18^F-FDG PET/CT	10	8
Perivascular or graft abnormal metabolic activity	6	8
Leukocyte scan	1	2
Vascular radiolabeled leukocyte uptake	0	1

^18^F-FDG PET/CT, ^18^F-fluoro-deoxyglucose-positron emission tomography/computed tomography.

### Microbiologic Diagnosis and Antimicrobial Susceptibility Profiles

**Figure 2:**
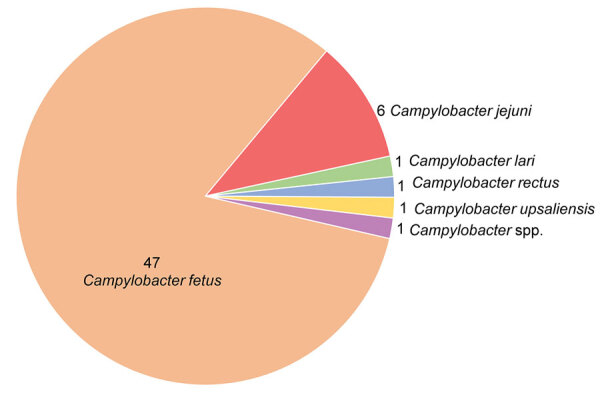
Distribution of *Campylobacter* species among 57 patients with vascular infections and endocarditis in a multicenter retrospective study on vascular infections and endocarditis caused by *Campylobacter* spp., France. Numbers indicate no. cases.

*C. fetus* was the most frequently identified species (82.5%), followed by *C. jejuni* (10.5%) (Figure 2). *C. fetus* was responsible for 92.3% of endocarditis and 80% of vascular infections. A *C. rectus* infection occurred on a gastroduodenal artery in a neutropenic patient with diabetes who was co-infected with commensal bacteria of the oral cavity. As comparison, among the initial cohort of patients with bacteremia, *C. jejuni* (42.9%) and *C. fetus* (42.6%) were the most commonly identified species, followed by *C. coli* (6.8%) and *C. ureolyticus* (3.7%) (*6*). Among the 252 patients with *C. fetus* bacteremia, 29 (11.5%) patients had vascular localization, and 11 (4.4.%) had endocarditis. However, secondary vascular localizations were not systematically researched and might have been underdiagnosed in the initial cohort.

Blood cultures were performed in all patients and were positive in 100% of patients with endocarditis and 88.9% of patients with vascular infections. The median time to positive blood samples was rather long, 55.5 hours (IQR 44.95–73 hours). The 5 patients with vascular infections and negative blood cultures were all infected by *C. fetus* species. Intraoperative specimens were positive in 11 vascular infections over the 14 cultures performed and 1 of 2 patients with endocarditis. Only 1 patient had a positive stool culture (*C. jejuni*) among the 12 performed.

We assessed antimicrobial-acquired resistance (Table 4). No strain was resistant to amoxicillin/clavulanate or to imipenem among the levels tested. Higher rates of resistance were observed for ampicillin (9.8%), fluoroquinolones (31.4%), and tetracycline (20.5%).

**Table 4:** Antimicrobial resistance by species of *Campylobacter* spp. identified in a multicenter retrospective study on vascular infections and endocarditis caused by *Campylobacter* spp., France*

Antimicrobial tested	MIC breakpoint, mg/L	No. (%) isolates		
		*C. fetus*	*C. jejuni*	Other *Campylobacter* spp.
Ampicillin	16	1/44 (2.3)	3/5 (60)	1/2 (50)
Amoxicillin/clavulanate	16	0/40	0/5	0/3
Ciprofloxacin	0.5	11/44 (25)	3/5 (60)	2/2 (100)
Erythromycin	4	1/43 (2.3)	0/5	0/2
Tetracycline	2	5/38 (13.2)	4/5 (80)	0/1
Gentamicin†	2	0/41	0/5	0/2
Imipenem	2	0/7	Not tested	0/1

*Strains susceptible to gentamicin were assumed to be susceptible to amikacin.

### Clinical Outcome

Survival without relapse at 3 months was observed for 67.3% (33/49) of the patients with available data at follow-up; the mortality rate was estimated at 24.5% (12 cases), and estimated relapse rate was 8.2% (4 cases). Two patients with endocarditis and 10 patients with vascular localization died within 3 months.

Among the 56 patients for whom antimicrobial therapy was documented, 54 (96.4%) received an appropriate treatment based on the susceptibility results. Only 2 patients were inefficiently treated (1 by third-generation cephalosporine, 1 by ofloxacin). Regarding antimicrobial therapy regimen among patients with VGEIs or prosthetic valves, 9/24 (37.5%) received single therapy and 15/24 (62.5%) dual therapy, combining a β-lactam with either a fluoroquinolone (9 patients), an aminoglycoside (5 patients), or a macrolide (1 patient). All patients with endocarditis received an initial association of aminoglycoside infusion and a β-lactam, except for 1 who received an aminoglycoside and fluoroquinolone therapy. The median duration of antimicrobial treatment was 42 days (IQR 20–49 days). Five patients received lifelong suppressive antimicrobial therapy (amoxicillin, amoxicillin/clavulanate, or ofloxacin).

We analyzed risks factors for 3-month mortality and relapse. Neither time to efficient therapy, immunosuppression, surgery, nor antimicrobial therapy regimen (single versus dual therapy) was associated with 3-month mortality in multivariate analysis. However, the time to efficient antimicrobial therapy initiation after the first symptoms was much longer in the patients who relapsed compared with relapse-free patients (61 days [IQR 20.3–104.3 days] vs. 9.5 days [IQR 2–15 days]; p = 0.006). Relapse patients also more often had diabetes (75% vs. 12%; p = 0.022) and osteoarticular-associated infection (75% vs. 2%; p = 0.001) than did relapse-free patients.

Nine of the 15 patients with VGEIs underwent surgery, and 7 underwent complete graft removal. Two patients with prosthetic valve endocarditis underwent surgery for valve replacement, and 2 underwent infected pacemaker replacement. Two more patients required surgery because of severe valve dysfunction.

## Discussion

We examined a comprehensive series of *Campylobacter* spp. cases associated vascular infections and endocarditis among 57 patients identified because of the participation of 37 hospitals in France. Consistent with data on *Campylobacter* spp. bacteremia, male and elderly patients were predominantly affected, and most patients had underlying conditions, particularly cardiovascular conditions, diabetes, solid neoplasm, chronic renal failure, or hepatic failure (*6*). Of interest, although *Campylobacter* spp. is the leading cause of bacterial diarrhea responsible for enteritis mainly occurring before 30 years of age in immunocompetent patients, invasive infections are more likely to affect immunocompromised elderly patients (*7*).

Native vascular infections preferentially affect the infra-renal aorta; *Salmonella* spp. and *Staphylococcus aureus* were the most commonly identified bacteria in previous studies (*14*,*15*). *Campylobacter* spp. involvement is rarely described, even though it represented almost 10% of infective native aortic aneurysms in a recent study in France (*16*). In our study, 66.7% of infections were aortic, and 15.6% occurred on peripheral arteries. Venous infections were also reported, but thrombophlebitis is poorly described so far because only a few case reports are available (*17*–*19*).

The 13 cases of endocarditis included in our study and the 21 case reports previously described in the literature highlight the role of this non-HACEK (species other than *Haemophilus* species, *Actinobacillus* *actinomycetemcomitans*, *Cardiobacterium* *hominis*, *Eikenella corrodens*, or *Kingella* spp.) gram-negative bacillus in endocarditis (Appendix). So far, the International Collaboration on Infective Endocarditis Prospective Cohort Study has described 49 (1.8%) endocarditis attributable to non-HACEK gram-negative bacilli among 2,761 patients with definite endocarditis (*20*). Most commonly encountered bacilli were *Escherichia coli* and *Pseudomonas aeruginosa,* but no *Campylobacter* spp. infection was reported. These gram-negative bacilli infections were severe, leading to an increased in-hospital mortality rate of 24% compared with 8% for *Streptococcus* spp. and 33% for *S. aureus*–associated endocarditis (*21*). In our study, the 3-month mortality rate associated with endocarditis was 15.4%, but it remains difficult to conclude given the small number of patients. Non-HACEK gram-negative bacilli endocarditis is usually associated with active injection drug use (up to 93% of cases) and therefore involves native tricuspid valve in most cases (*22*). In our cohort, all endocarditis cases were left-sided, and 69.2% occurred either on prosthetic valves or intracardiac devices. This profile looks more like other foodborne endocarditis, such as *Salmonella* spp. or *Listeria monocytogenes*, which more likely affect older and immunocompromised patients and are associated with higher mortality rates (42.5% for *Salmonella* spp. [*23*] and 41% for *L. monocytogenes* [*24*]).

*C. fetus* was the most frequently involved species (82.5%), whereas according to the report of NRCCH, this species represents only 1% of the isolates analyzed among the 8,082 isolates received in 2020 (*25*). In our study, the most frequently isolated species are *C. jejuni* (84%) and *C. coli* (13%), and they both are predominantly isolated from stool culture (98.8% of *C. jejuni* and 99.6% of *C. coli*). *C. fetus* is much less common (1%) and is predominantly isolated from blood culture or deep samplings (57%) compared with stool samples (41%) (*25*). *C. fetus* virulence is notably attributable to a protein capsule called the S-layer, which impairs complement activation by a lack of C3b binding (*26*). Different mechanisms have been suggested to explain its tropism for vascular endothelium, especially when the latter is previously damaged, such as the production of procoagulant factors favoring the formation of microthrombi or the presence of a membrane receptor with an affinity for the endothelium (*27*).

Regarding the nonspecific symptoms, because only 22.8% of patients had diarrhea, and because of the lack of awareness of the risk for secondary localizations associated with campylobacteriosis, those complications might be underestimated. Among patients with *C. fetus* bacteremia in the campylobacteremia study, 11.5% had a vascular infection and 4.4% had endocarditis. Furthermore, 11% of the 99 patients who underwent echocardiography had endocarditis, close to the rate described for *S. aureus* bacteremia (*6*,*28*). Those findings warrant the use of systematic transthoracic echocardiography in cases of *C. fetus* bacteremia. Foreign implants and preexisting aneurysms also seemed to be risk factors for bacterial colonization because vascular infections occurred on (endo)grafts in 33.3% of cases and native aneurysms in 24.4%. Therefore, a systematic computed tomography angiogram should be discussed for these patients. Moreover, the risk for 3-month relapse was associated with osteoarticular involvements, highlighting the paramount importance of a comprehensive diagnosis and treatment of these secondary localizations.

The association of 3-month relapse with delayed initiation of efficient antimicrobial therapy advocates for the necessity of prompt appropriate treatment. The retrospective design of our study did not enable us to make a conclusion on the optimal treatment modality. Nevertheless, considering low acquired resistance rates, which is consistent with NRCCH reports in recent years (*25*), an initial dual bactericidal therapy by amoxicillin/clavulanate and gentamicin could be a good option for empiric therapy. The issue of secondary targeted therapy remains unresolved. Amoxicillin and macrolides could be good options according to the susceptibility profile. Ciprofloxacin could also be of interest; however, the use of fluoroquinolones remains debated given the recent divergent data published on possible excess risks of aneurysmal rupture and aortic dissection (*29*,*30*).

*Campylobacter* spp. cardiovascular infections are rare but should be considered seriously in light of the high incidence of campylobacteriosis. These infections are associated with high mortality rates and mainly occur in elderly patients with underlying conditions. The relapse rate is also high and correlates with delayed initiation of an efficient antimicrobial therapy, suggesting a need for prompt recognition and treatment. Therefore, systematic transthoracic echocardiography should be performed in cases of *C. fetus* bacteremia. Dedicated imaging might also be indicated for patients with a preexisting aneurysm or vascular (endo)graft, even in the absence of evocative symptoms.

Appendix Additional information about multicenter retrospective study of vascular infections and endocarditis caused by *Campylobacter* spp., France.

## References

[1] Kaakoush NO, Castaño-Rodríguez N, Mitchell HM, Man SM. Global epidemiology of *Campylobacter* infection. Clin Microbiol Rev. 2015;28:687–720. 10.1128/CMR.00006-15 26062576 PMC4462680

[2] Tack DM, Marder EP, Griffin PM, Cieslak PR, Dunn J, Hurd S, et al. Preliminary incidence and trends of infections with pathogens transmitted commonly through food—Foodborne Diseases Active Surveillance Network, 10 U.S. sites, 2015–2018. MMWR Morb Mortal Wkly Rep. 2019;68:369–73. 10.15585/mmwr.mm6816a2 31022166 PMC6483286

[3] European Food Safety Authority; European Centre for Disease Prevention and Control. The European Union One Health 2020 zoonoses report. EFSA J. 2021;19:e06971. 36329690 10.2903/j.efsa.2021.6971 PMC9624447

[4] Gazaigne L, Legrand P, Renaud B, Bourra B, Taillandier E, Brun-Buisson C, et al. *Campylobacter fetus* bloodstream infection: risk factors and clinical features. Eur J Clin Microbiol Infect Dis. 2008;27:185–9. 10.1007/s10096-007-0415-0 17999095

[5] Feodoroff B, Lauhio A, Ellström P, Rautelin H. A nationwide study of *Campylobacter jejuni* and *Campylobacter coli* bacteremia in Finland over a 10-year period, 1998-2007, with special reference to clinical characteristics and antimicrobial susceptibility. Clin Infect Dis. 2011;53:e99–106. 10.1093/cid/cir509 21921217 PMC3174097

[6] Tinévez C, Velardo F, Ranc AG, Dubois D, Pailhoriès H, Codde C, et al.; Campylobacteremia study group. Retrospective multicentric study on *Campylobacter* spp. bacteremia in France: the Campylobacteremia Study. Clin Infect Dis. 2022;75:702–9. 10.1093/cid/ciab983 34849656

[7] Pacanowski J, Lalande V, Lacombe K, Boudraa C, Lesprit P, Legrand P, et al.; CAMPYL Study Group. *Campylobacter* bacteremia: clinical features and factors associated with fatal outcome. Clin Infect Dis. 2008;47:790–6. 10.1086/591530 18699745

[8] Fernández-Cruz A, Muñoz P, Mohedano R, Valerio M, Marín M, Alcalá L, et al. *Campylobacter* bacteremia: clinical characteristics, incidence, and outcome over 23 years. Medicine (Baltimore). 2010;89:319–30. 10.1097/MD.0b013e3181f2638d 20827109

[9] Habib G, Lancellotti P, Antunes MJ, Bongiorni MG, Casalta JP, Del Zotti F, et al.; ESC Scientific Document Group. 2015 ESC guidelines for the management of infective endocarditis. Eur Heart J. 2015;36:3075–128. 10.1093/eurheartj/ehv319 26320109

[10] Wilson WR, Bower TC, Creager MA, Amin-Hanjani S, O’Gara PT, Lockhart PB, et al.; American Heart Association Committee on Rheumatic Fever, Endocarditis, and Kawasaki Disease of the Council on Cardiovascular Disease in the Young; Council on Cardiovascular and Stroke Nursing; Council on Cardiovascular Radiology and Intervention; Council on Cardiovascular Surgery and Anesthesia; Council on Peripheral Vascular Disease; and Stroke Council. Vascular graft infections, mycotic aneurysms, and endovascular infections: a scientific statement from the American Heart Association. Circulation. 2016;134:e412–60. 10.1161/CIR.0000000000000457 27737955

[11] Chakfé N, Diener H, Lejay A, Assadian O, Berard X, Caillon J, et al.; Esvs Guidelines Committee. European Society for Vascular Surgery (ESVS) 2020 clinical practice guidelines on the management of vascular graft and endograft infections. Eur J Vasc Endovasc Surg. 2020;59:339–84. 10.1016/j.ejvs.2019.10.016 32035742

[12] European Committee on Antimicrobial Susceptibility Testing. Breakpoint tables for interpretation of MICs and zone diameters [cited 2022 Sep 1]. https://www.eucast.org/fileadmin/src/media/PDFs/EUCAST_files/Breakpoint_tables/v_12.0_Breakpoint_Tables.pdf

[13] Bessède E, Solecki O, Sifré E, Labadi L, Mégraud F. Identification of *Campylobacter* species and related organisms by matrix assisted laser desorption ionization-time of flight (MALDI-TOF) mass spectrometry. Clin Microbiol Infect. 2011;17:1735–9. 10.1111/j.1469-0691.2011.03468.x 21375659

[14] Zhu C, Zhao J, Huang B, Yuan D, Yang Y, Wang T. Long-term outcome of endovascular aortic repair for mycotic abdominal aortic aneurysm. ANZ J Surg. 2020;90:1376–80. 10.1111/ans.16122 32648327

[15] Heinola I, Sörelius K, Wyss TR, Eldrup N, Settembre N, Setacci C, et al. Open repair of mycotic abdominal aortic aneurysms with biological grafts: an international multicenter study. J Am Heart Assoc. 2018;7:e008104. 10.1161/JAHA.117.008104 29886419 PMC6220543

[16] Journeau L, de la Chapelle M, Guimard T, Ferfar Y, Saadoun D, Mahé I, et al. A strobe multicenter descriptive study of 55 infectious aortitis. Medicine (Baltimore). 2020;99:e22422. 10.1097/MD.0000000000022422 33019420 PMC7535642

[17] Jawad II, Chandna A, Morris-Jones S, Logan S. Unusual case of Lemierre’s syndrome. BMJ Case Rep. 2018;11:e226948. 10.1136/bcr-2018-226948 30567118 PMC6301635

[18] Morrison VA, Lloyd BK, Chia JKS, Tuazon CU. Cardiovascular and bacteremic manifestations of *Campylobacter fetus* infection: case report and review. Rev Infect Dis. 1990;12:387–92. 10.1093/clinids/12.3.387 2193344

[19] Carbone KM, Heinrich MC, Quinn TC. Thrombophlebitis and cellulitis due to *Campylobacter fetus* ssp. fetus. Report of four cases and a review of the literature. Medicine (Baltimore). 1985;64:244–50. 10.1097/00005792-198507000-00005 3892221

[20] Morpeth S, Murdoch D, Cabell CH, Karchmer AW, Pappas P, Levine D, et al.; International Collaboration on Endocarditis Prospective Cohort Study (ICE-PCS) Investigators. Non-HACEK gram-negative bacillus endocarditis. Ann Intern Med. 2007;147:829–35. 10.7326/0003-4819-147-12-200712180-00002 18087053

[21] Hill EE, Herijgers P, Claus P, Vanderschueren S, Herregods MC, Peetermans WE. Infective endocarditis: changing epidemiology and predictors of 6-month mortality: a prospective cohort study. Eur Heart J. 2007;28:196–203. 10.1093/eurheartj/ehl427 17158121

[22] Veve MP, McCurry ED, Cooksey GE, Shorman MA. Epidemiology and outcomes of non-HACEK infective endocarditis in the southeast United States. PLoS One. 2020;15:e0230199. 10.1371/journal.pone.0230199 32155223 PMC7064227

[23] Cheng WL, Li CW, Li MC, Lee NY, Lee CC, Ko WC. *Salmonella* infective endocarditis. J Microbiol Immunol Infect. 2016;49:313–20. 10.1016/j.jmii.2015.02.659 25882489

[24] Shoai-Tehrani M, Pilmis B, Maury MM, Robineau O, Disson O, Jouvion G, et al.; Listeria endovascular infections study group. *Listeria monocytogenes*-associated endovascular infections: A study of 71 consecutive cases. J Infect. 2019;79:322–31. 10.1016/j.jinf.2019.07.013 31376457

[25] Centre National de Référence des Campylobacters et Hélicobacters. Rapport annuel d’activité 2021 Centre National de Référence des Campylobacters et des Hélicobacters année d’exercice 2020 [cited 2022 Sep 01]. https://www.cnrch.fr/bilans-et-publications/bilans-annuels-cnr-ch

[26] Sprenger H, Zechner EL, Gorkiewicz G. So close and yet so far - Molecular Microbiology of *Campylobacter fetus* subspecies. Eur J Microbiol Immunol (Bp). 2012;2:66–75. 10.1556/EuJMI.2.2012.1.10 24611123 PMC3933992

[27] Wagenaar JA, van Bergen MAP, Blaser MJ, Tauxe RV, Newell DG, van Putten JPM. *Campylobacter fetus* infections in humans: exposure and disease. Clin Infect Dis. 2014;58:1579–86. 10.1093/cid/ciu085 24550377 PMC10942002

[28] Le Moing V, Alla F, Doco-Lecompte T, Delahaye F, Piroth L, Chirouze C, et al.; VIRSTA study group. *Staphylococcus aureus* bloodstream infection and endocarditis—a prospective cohort study. PLoS One. 2015;10:e0127385. 10.1371/journal.pone.0127385 26020939 PMC4447452

[29] Dong YH, Chang CH, Wang JL, Wu LC, Lin JW, Toh S. Association of infections and use of fluoroquinolones with the risk of aortic aneurysm or aortic dissection. JAMA Intern Med. 2020;180:1587–95. 10.1001/jamainternmed.2020.4192 32897358 PMC7489369

[30] Newton ER, Akerman AW, Strassle PD, Kibbe MR. Association of fluoroquinolone use with short-term risk of development of aortic aneurysm. JAMA Surg. 2021;156:264–72. 10.1001/jamasurg.2020.6165 33404647 PMC7788511

