# Correction: Vol. 29, No. 3

**DOI:** 10.3201/eid2911.C12911

**Published:** 2023-11

**Keywords:** errata, erratum, correction

The author order was incorrect in Multicenter Retrospective Study of Vascular Infections and Endocarditis Caused by *Campylobacter* spp., France (Tinévez C et al.). The article has been corrected online (https://wwwnc.cdc.gov/eid/article/29/3/22-1417_article).

