# Supplementary material for: Multicenter Retrospective Study of Vascular Infections and Endocarditis Caused by Campylobacter spp., France
Source: Emerg Infect Dis. 2023 Mar;29(3):484–92. doi: 10.3201/eid2903.221417 (PMC9973684; doi:10.3201/eid2903.221417)
Supplement: Appendix — Additional information about multicenter retrospective study of vascular infections and endocarditis caused by Campylobacter spp., France. [file 22-1417-Techapp-s1.pdf]

Article DOI: https://doi.org/10.3201/eid2903.221417

Multicenter Retrospective Study of Vascular Infections and Endocarditis Caused by *Campylobacter* spp., France

Appendix

Appendix Table. Literature review of endocarditis due to *Campylobacter* spp.*

Case	Sex	Age	Localization	Species	Ref
1	F	60	Aortic valve	*Campylobacter fetus*	(*1*)
2	M	75	Aortic valve	*C. fetus*	(*2*)
3	M	85	Mitral valve	*C. fetus*	(*3*)
4	M	51	Tricuspid valve	*C. fetus*	(*4*)
5	M	91	Aortic valve	*C. fetus*	(*5*)
6	M	52	Tricuspid valve	*C. fetus*	(*6*)
7	M	49	Tricuspid valve	*C. fetus*	(*7*)
8	M	44	Aortic valve	*C. fetus*	(*8*)
9	H	41	Aortic valve	*Campylobacter jejuni*	(*9*)
10	M	48	Tricuspid valve	*C. fetus*	(*10*)
11	F	85	Prosthetic aortic valve	*C. fetus*	(*11*)
12	M	50	Aortic valve	*C. jejuni*	(*12*)
13	M	70	Aortic valve	*C. fetus*	(*13*)
14	M	18	Aortic valve	*C. fetus*	(*14*)
15	F	76	Prosthetic aortic valve	*C. fetus*	(*1*)
16	M	70	Prosthetic aortic valve	*C. fetus*	(*15*)
17	M	61	Prosthetic aortic valve	*C. fetus*	(*16*)
18	M	65	Pace maker	*C. fetus*	(*17*)
19	F	84	Prosthetic aortic valve	*C. fetus*	(*18*)
20	F	48	Prosthetic mitral valve	*C. fetus*	(*19*)
21	M	65	Post-Bentall graft	*C. fetus*	(*20*)

*A systematic review was conducted. Screened studies were case report written in English, fully accessible and exhaustive. Searching was performed in Pubmed, EMBASE and Google Scholar databases.

References

1. Farrugia DC, Eykyn SJ, Smyth EG. *Campylobacter fetus* endocarditis: two case reports and review. Clin Infect Dis. 1994;18:443–6. PubMed https://doi.org/10.1093/clinids/18.3.443

2. Suy F, Le Dû D, Roux AL, Hanachi M, Dinh A, Crémieux AC. Meningitis and endocarditis caused by *Campylobacter fetus* after raw-liver ingestion. J Clin Microbiol. 2013;51:3147–50. PubMed https://doi.org/10.1128/JCM.00631-13

3. Rollot K, Albert JD, Werner S, Tattevin P, Cozic I, Perdriger A, et al. *Campylobacter fetus* septic arthritis revealing a malignancy. Joint Bone Spine. 2004;71:63–5. PubMed https://doi.org/10.1016/S1297-319X(03)00094-0

4. Mahe I, Perdrix C, Maniere T, Holeman A, Diemer M, Bergmann JF. A case of *Campylobacter fetus* endocarditis of the tricuspid valve unaccompanied by fever. Am J Med. 2001;111:418. PubMed https://doi.org/10.1016/S0002-9343(01)00874-9

5. Baty V, Hoen B, Selton-Suty C, Schuhmacher H, Peiffert B, Danchin N, et al. *Campylobacter fetus* endocarditis manifested by a popliteal mycotic aneurysm [in French]. Presse Med. 1998;27:357–8. PubMed

6. Miki K, Maekura R, Hiraga T, Hirotani A, Hashimoto H, Kitada S, et al. Infective tricuspid valve endocarditis with pulmonary emboli caused by *Campylobacter fetus* after tooth extraction. Intern Med. 2005;44:1055–9. PubMed https://doi.org/10.2169/internalmedicine.44.1055

7. Ohshima H, Itaoka Y, Ohno M, Hara K, Kashida M, Kuwako K, et al. A case of right-sided infective endocarditis in a drug addict [in Japanese]. Kokyu To Junkan. 1990;38:277–81. PubMed

8. Wozniak R, Middleton J, Chmel H, de la Cruz C, Young R. Gram-negative endocarditis caused by *Campylobacter fetus*. South Med J. 1978;71:1311–2. PubMed https://doi.org/10.1097/00007611-197810000-00032

9. Bucknell SJ, Le T, Amerena J, Hill DG, McDonald M. Aortic dissection associated with *Campylobacter aortitis*. Heart Lung Circ. 2000;9:88–91. PubMed https://doi.org/10.1046/j.1444-2892.2000.00027.x

10. Sasaki Y, Suehiro S, Shibata T, Minamimura H, Kumano H, Kinoshita H. A case of isolated tricuspid valve endocarditis caused by *Campylobacter fetus* [in Japanese]. Nihon Kyobu Geka Gakkai Zasshi. 1997;45:1844–7. PubMed

11. Petridou C, Strakova L, Simpson R. *Campylobacter fetus* prosthetic valve endocarditis presenting as a stroke. JMM Case Rep. 2018;5:e005147. PubMed https://doi.org/10.1099/jmmcr.0.005147

12. Sitter T, Bauer MF, Held E. Acute aortic insufficiency following endocarditis due to infection with *Campylobacter fetus* subspecies fetus [in German]. Dtsch Med Wochenschr. 1992;117:1355–8. PubMed https://doi.org/10.1055/s-2008-1062450

13. Allerberger F, Kasten MJ, Anhalt JP. *Campylobacter fetus* subspecies fetus infection. Klin Wochenschr. 1991;69:813–6. PubMed https://doi.org/10.1007/BF01744276

14. Usui N, Sakai K, Muraguchi T, Tsukamoto Y, Kimura E, Nishizawa K, et al. Cases of infective endocarditis caused by *Campylobacter fetus*. Osaka City Med J. 1989;35:63–9. PubMed

15. Reid MJA, Shannon EM, Baxi SM, Chin-Hong P. Steak tartare endocarditis. BMJ Case Rep. 2016:bcr2015212928. http://dx.doi.org/10.1136/bcr-2015-212928 **PMID: 26917729**

16. Peetermans WE, De Man F, Moerman P, van de Werf F. Fatal prosthetic valve endocarditis due to *Campylobacter fetus*. J Infect. 2000;41:180–2. PubMed https://doi.org/10.1053/jinf.2000.0699

17. Sękowska A, Fabiszak T, Mikucka A, Andrzejewska M, Kruszyńska E, Gospodarek E, et al. A case of defibrillator-associated infective endocarditis due to *Campylobacter fetus*. Folia Microbiol (Praha). 2016;61:529–32. PubMed https://doi.org/10.1007/s12223-016-0466-7

18. Désidéri-Vaillant C, Guichon J-M, Noyer V, Nedelec Y, Galinat H, Sapin-Lory J, et al. *Campylobacter fetus* endocarditis: a case report [in French]. Ann Biol Clin (Paris). 2013;71:465–7. https://doi.org/10.1684/abc.2013.0862 **PMID: 23906576**

19. Caramelli B, Mansur AJ, Grinberg M, Mendes CM, Pileggi F. *Campylobacter fetus* endocarditis on a prosthetic heart valve. South Med J. 1988;81:802–3. PubMed https://doi.org/10.1097/00007611-198806000-00028

20. Haruyama A, Toyoda S, Kikuchi M, Arikawa T, Inami S, Otani N, et al. *Campylobacter fetus* as cause of prosthetic valve endocarditis. Tex Heart Inst J. 2011;38:584–7. PubMed
